# A perfluorocyclopentene based diarylethene bearing two terpyridine moieties – synthesis, photochemical properties and influence of transition metal ions

**DOI:** 10.3762/bjoc.6.53

**Published:** 2010-05-26

**Authors:** Falk Wehmeier, Jochen Mattay

**Affiliations:** 1 Institut für Chemie, Humboldt-Universität zu Berlin, Brook-Taylor-Str. 2, D-12489 Berlin, Germany; 2 Department of Chemistry, Organic Chemistry 1, Bielefeld University, P.O. Box 10 01 31, D-33501 Bielefeld, Germany

**Keywords:** coordination chemistry, diarylethene, photochromism, terpyridine

## Abstract

The synthesis of a perfluorocyclopentene based diarylethene bearing two terpyridine units is reported. Furthermore studies of the free ligand’s photochromism and investigations regarding the influence of various transition metal ions on the photochromic reaction are presented. The photochromism of the central diarylethene unit is strongly dependent on the transition metal present, vice versa the photochromic reaction seems to influence the MLCT transition of a binuclear Ru(II) complex.

## Introduction

2,2′:6′,2″-Terpyridines have been of great interest over the last years, mostly because of their ability to chelate transition metals. The special (photochemical) properties of their metal complexes have led to the development of various luminescent metal compounds [1] and sensitizers for photovoltaic devices [2–3]. Ditopic terpyridyl units have been recently used to develop electrochemical sensors [4–5]. A microreview concerning the synthesis of functionalized terpyridines has also been published since the electronic properties of the ligand are influenced by the substituents present [6].

Because of this impact of terpyridine derivatives in photochemistry, we focused our attention on the synthesis and studies of photoswitchable terpyridine ligands. The synthesis of bisterpyridines linked by a diazogroup has been reported [7–8], as well as the connection of terpyridines to spiropyran moieties [9]. There have also been recent reports about terpyridines linked to dithienylethenes [10–11]. Herein we report the synthesis of terpyridine functionalized diarylethenes based on perfluorocyclopentene, their photochemistry and investigations regarding the influence of transition metals.

## Results and Discussion

### Synthetic key steps

**Scheme 1:**
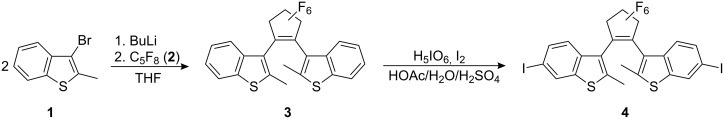
Synthesis of twofold iodinated bis(benzo[*b*]thiophenyl)perfluorocyclopentene **4**.

The basic photochromic diarylethene unit (**3**) can be obtained by lithiation of 3-bromo-2-methylbenzo[*b*]thiophene (**1**) followed by quenching with perfluorocyclopentene (**2**), according to the method described by Irie [12]. Electrophilic iodination leads to the diiodo photoswitch **4** (Scheme 1) [13–14].

**Scheme 2:**
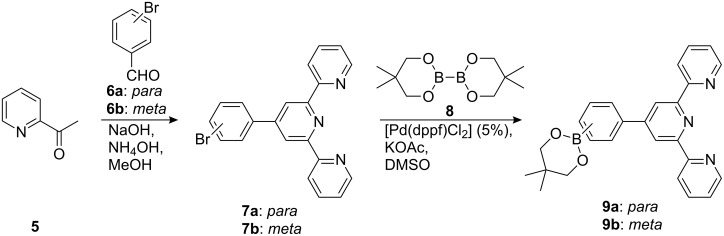
Synthesis of terpyridinyl boronic acids **9a** and **9b**.

We decided to use the diiodo switch **4** for catalytic cross coupling reactions, since approaches to a direct terpyridine synthesis, starting from suitable diarylethene aldehydes (Kröhnke condensation), were unsuccessful. Moreover, the diiodo switch **4** has been previously used for Suzuki-type cross coupling reactions [14]. Synthetic routes to terpyridine substituted diarylethenes via the above mentioned catalytic cross coupling reactions require suitably functionalized terpyridine precursors, e.g. boronic acid derivatives for Suzuki type cross coupling reactions. 4′-(4-Bromophenyl)-2,2′:6′,2″-terpyridine (**7a**) and its *meta*-substituted analogue **7b** can be synthesized by Kröhnke-condensation of 2-acetylpyridine (**5**) with *p*-bromobenzaldehyde (**6a**) or *m*-bromobenzaldehyde (**6b**), respectively [15]. Miyaura type cross coupling reactions of **7a** and **7b** with 5,5,5′,5′-tetramethyl-2,2′-di(1,3,2-dioxaborinan) (**8**) led to the formation of boronic acid derivatives **9a** [16] and **9b** (Scheme 2).

**Scheme 3:**
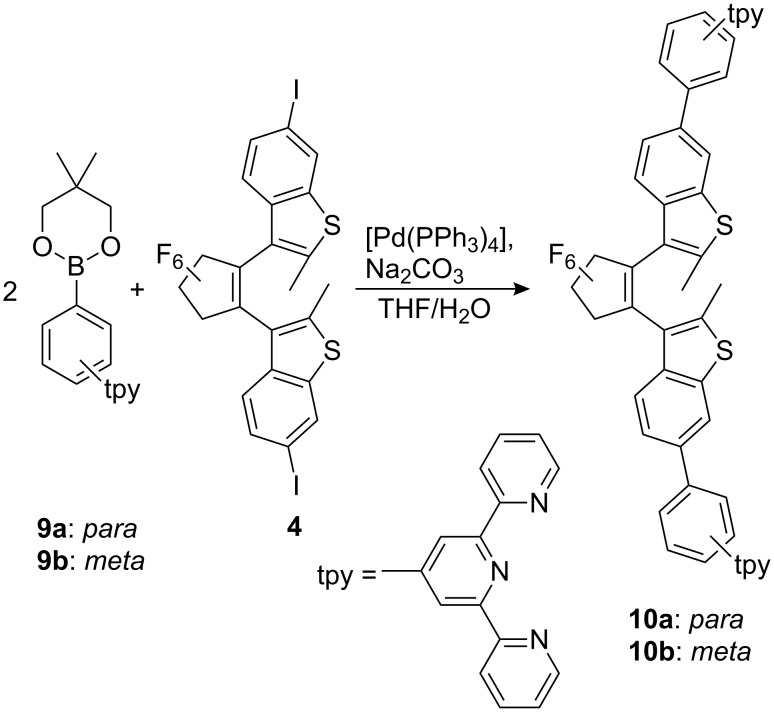
Synthesis of the bis(terpyridinyl)diarylethenes **10a** and **10b**.

The terpyridine moieties can be attached to the diarylethene unit by Suzuki type cross coupling of the diiodo switch **4** with the boronic esters **9a** and **9b** under conditions similar to those described for other aryl boronic acids and their derivatives to yield the target molecules **10a** and **10b** (Scheme 3) [14].

Whilst **10a** can be purified by column chromatography, all efforts to obtain highly pure *meta*-substituted **10b** have, so far, been unsuccessful – MALDI-TOF-measurements nevertheless indicate the formation of **10b**, and the proton-NMR spectrum displays the expected signals (see Experimental Section and Supporting Information File 1).

### Photochromism of the free ligand

**Scheme 4:**
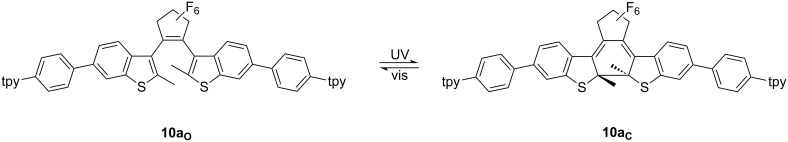
Photochromic reaction of the free ligand **10a**.

The obtained terpyridine substituted diarylethene **10a** shows the anticipated photochromic behavior, undergoing photocyclization under irradiation with UV-light (Scheme 4).

**Figure 1:**
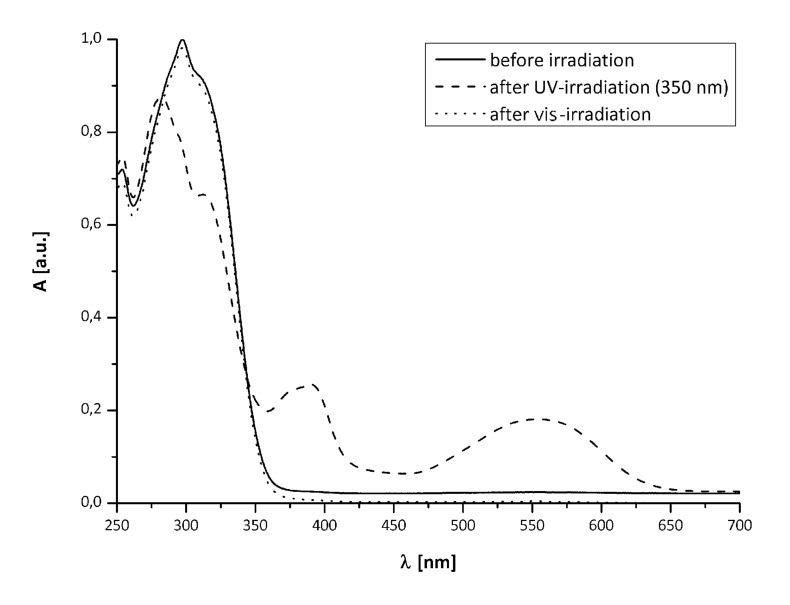
UV–vis-spectra of **10a** before (*solid*), after UV-irradiation (*dashed*) and after irradiation with vis light (*dotted*).

Figure 1 shows the absorption spectra of **10a** (*solid line*), **10a_C_** (*dashed line*, formed by irradiation with λ = 350 nm) and after re-opening with vis light (*dotted line*).

### Influence of transition metals

#### A binuclear Ru(II)-complex

**Scheme 5:**
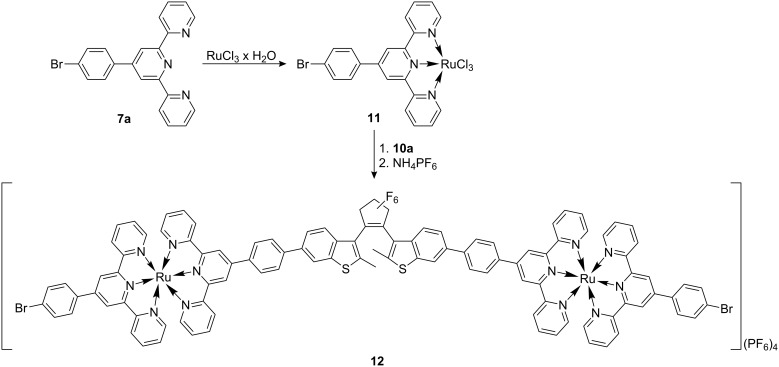
Synthesis of the binuclear Ru(II)-complex **12**.

For investigations of the photochemical behavior of **10a** in the coordination sphere of ruthenium(II), we synthesized the binuclear complex **12**, from ruthenium(III) chloride hydrate via the monoterpyridine complex **11** (Scheme 5).

**Figure 2:**
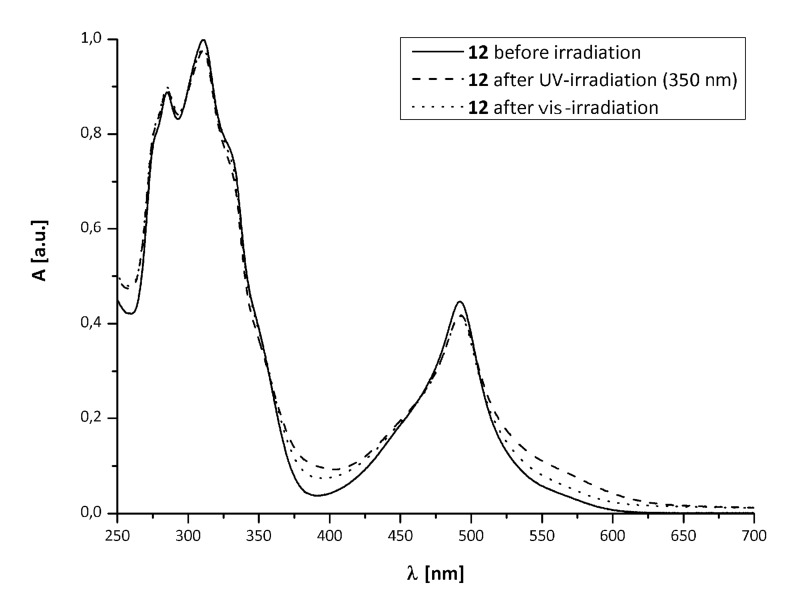
UV–vis-spectra of **12** before (*solid*), after UV-irradiation (*dashed*) and after irradiation with vis light (*dotted*).

The UV–vis-spectra of the binuclear complex **12** in acetonitrile solution are shown in Figure 2 – before (*solid*), after UV-irradiation (*dashed*) and after irradiation with vis light (*dotted*).

**Figure 3:**
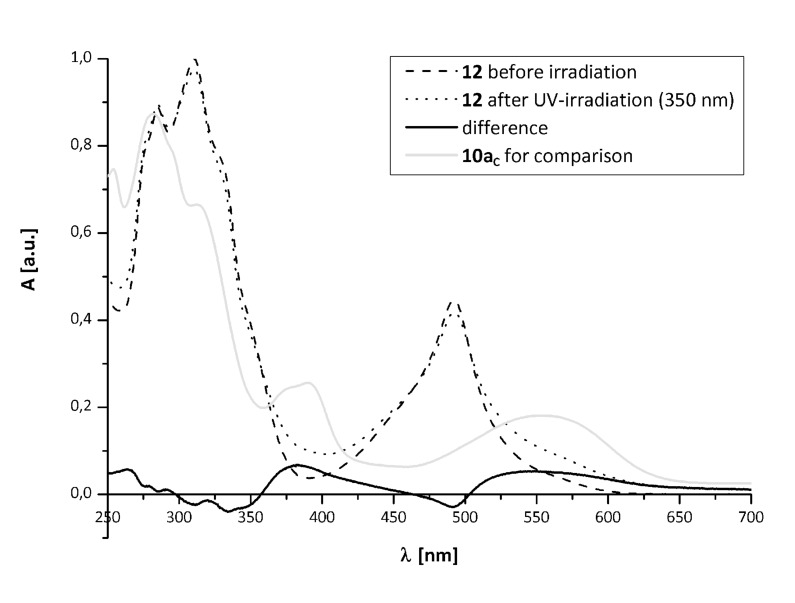
UV–vis-spectra of **12** before (*dashed*), after UV-irradiation (*dotted*), the difference (*solid*) and free **10a_C_** for comparison (*grey*).

The absorption of the strong MLCT band at λ = 490 nm decreases upon UV-irradiation, while absorption in the visible region of the spectrum increases. This may be regarded as indication that the photochromic reaction of the ligand not only takes place in presence of ruthenium, but also influences the MLCT transition. This supposition is supported by the difference spectra (Figure 3, the absorption of the free ligand **10a_C_** is shown in grey for comparison).

According to studies with similar ruthenium(II) complexes of terpyridine functionalized dithienylethenes [10], the intensity of the MLCT band can be attributed to communication between the ruthenium nuclei. In this case communication is obviously reduced by the bridging photoreaction of the diarylethene [9]. On one hand this seems quite unexpected, since the extended π-system of the closed isomer **10a_C_** should facilitate charge transfer processes between the ruthenium nuclei. On the other hand the closed isomer **10a_C_** might act as an acceptor; a similar effect has been observed with a spiropyran moiety bridging two terpyridine units, whose (closed) merocyanine form inhibits the energy transfer between metal centres and thus acts as a T-junction relay [9].

#### Influence of other transition metal ions in solution

In the following photochemical investigations we added various transition metal ions to methanolic suspensions of the bisterpyridine **10a** to generate terpyridine-complexes in situ [17]. Table 1 shows the results obtained with Fe(II), Co(II), Ni(II) and Zn(II).

**Table 1:** Influence of different 3d-transition metals on the photochromic reaction of **10a** in solution.

Transition metal ion (precursor)	Photochromic reaction

Fe^2+^ (FeCl_2_ × 4 H_2_O)	No reaction^a^
Co^2+^ (CoCl_2_)	Slow reaction^b^
Ni^2+^ ([Ni(acac)_2_])	Slow reaction^b^
Zn^2+^ (Zn(OTf)_2_)	Fast reaction^c^

^a^Figure 4. ^b^See Supporting Information File 1. ^c^Figure 5.

**Figure 4:**
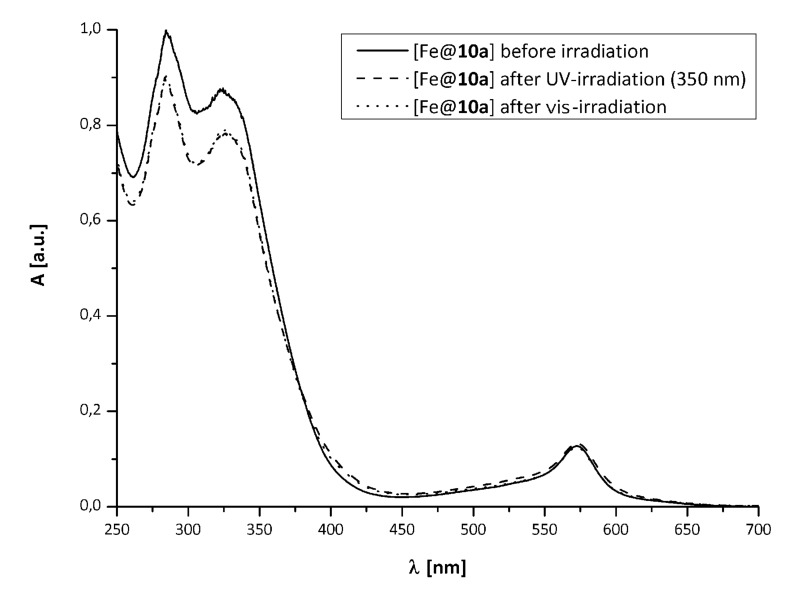
UV–vis-spectra of [Fe^2+^@**10a**] before (*solid*), after UV-irradiation (*dashed*) and after irradiation with vis light (*dotted*).

The UV–vis-spectra of the iron(II) complex [Fe^2+^@**10a**], obtained by reaction of **10a** with ferric chloride tetrahydrate, are shown in Figure 4.

Apart from the expected bathochromic shift of the UV-bands of the ligand [18], an MLCT band at λ = 570 nm was observed. While the UV absorption decreases upon UV-irradiation, no significant change of the absorption in the visible region occurred. This indicates inhibition of the photochromic reaction of the diarylethene by the MLCT transition, as previously reported for other iron(II) complexes of bisterpyridine thienylethenes [10].

In contrast to the iron(II) complex, the ditopic ligand seems to retain its photochromic properties in the cobalt(II) and nickel(II) complexes – synthesized from CoCl_2_ and [Ni(acac)_2_], respectively – although the photocyclisation takes much longer than with free **10a** (about 5 minutes compared to ca. 30 seconds). This observation may be due to side processes involving charge transfer transitions at the transition metal centres.

**Figure 5:**
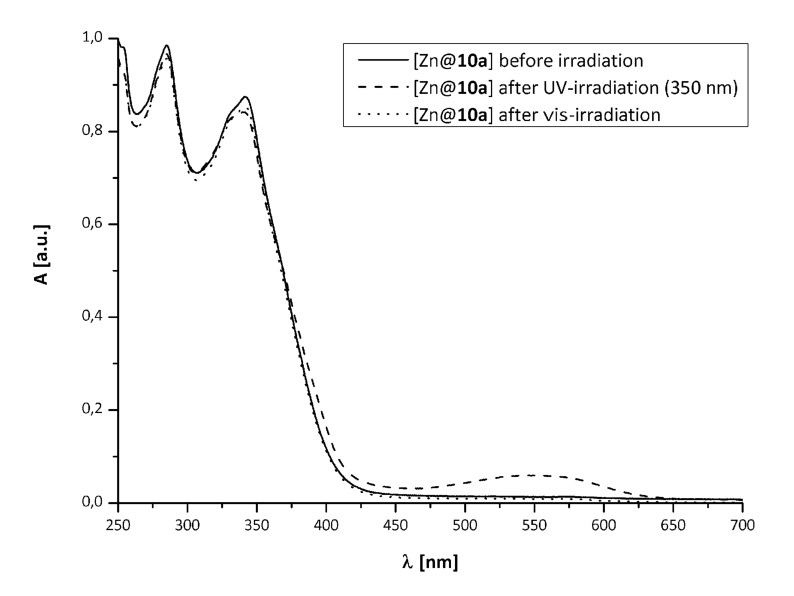
UV–vis-spectra of [Zn^2+^@**10a**] before (*solid*), after UV-irradiation (*dashed*) and after irradiation with vis light (*dotted*).

The corresponding zinc(II) complex [Zn^2+^@**10a**] was synthesized from zinc(II) trifluoromethansulfonate and **10a** in methanol. The UV–vis-spectra are shown in Figure 5.

In this case neither inhibition nor any significant slowing of the photoreaction of the ligand was observed.

## Conclusion

We have developed the synthesis of two highly fatigue resistant bis(terpyridinyl) diarylethenes by Suzuki cross coupling methods. The photochemical behavior of the free ligand **10a** met our expectations regarding reversibility. It was shown that the presence of various transition metal ions significantly influences the photochromism of the bridging unit: While iron(II)-ions completely inhibit the photochromic reaction, cobalt(II) and nickel(II) appear to slow down the photoreaction considerably. Zinc(II)-ions, on the other hand, had no influence on the photochromism of the central diarylethene unit. A rather special case is the binuclear ruthenium(II) complex **12**: The diarylethene seems to undergo the expected photochromic reaction, but at the same time the absorption intensity of the MLCT band of the complex decreased, indicating a diminution in communication between the metal centres. The synthesis and investigation of asymmetrical complexes such as [L_n_Os(*tpy-diae-tpy*)RuL_n_] should provide further insight into the role the diarylethene unit plays in the ditopic ligand.

## Experimental

**General**: *p*-Bromobenzaldehyde (**6a**), *m*-bromobenzaldehyde (**6b**) and 5,5,5′,5′-tetramethyl-2,2′-di(1,3,2-dioxaborinan) (**8**) are commercially available and were used as supplied. Perfluorocyclopentene (**2**) was a generous gift from Masahiro Irie (see Acknowledgements). 2-Acetylpyridine (**5**) was distilled before use. 3,3′-(Perfluorocyclopent-1-ene-1,2-diyl)bis(2-methylbenzo[*b*]thiophene) (**3**) [12], 3,3′-(perfluorocyclopent-1-ene-1,2-diyl)bis(6-iodo-2-methylbenzo[*b*]thiophene) (**4**) [13–14], 4′-(4-bromophenyl)-2,2′:6′,2″-terpyridine (**7a**) [15] and 4′-[4-(5,5-dimethyl-1,3,2-dioxaborinan-2-yl)phenyl]-2,2′:6′,2″-terpyridine (**9a**) [16] were synthesized according to previously reported procedures. Solvents and chemicals were dried by standard methods.

Irradiation experiments were carried out in a quartz cuvette (d = 1 cm) and the solutions were not degassed before irradiation. UV-spectra were recorded with a Lambda 40 (Perkin-Elmer) at room temperature.

NMR-spectra were recorded with a Bruker DRX 500. EI-mass spectra were recorded with an Autospec X (Vacuum Generators), ESI-mass spectra were recorded with a Bruker Esquire 3000; high resolution-mass spectra were recorded with a Bruker Apex III-FT-ICR. Measured and calculated masses are true ion masses, taking into account the mass of lost (or added) electrons.

### Synthesis of 4′-(3-bromophenyl)-2,2′:6′,2″-terpyridine (**7b**)

1.5 g (8 mmol) *m*-Bromobenzaldehyde (**6b**) was dissolved in 220 mL methanol followed by the addition of 2.0 g (16 mmol) freshly distilled 2-acetylpyridine (**5**) and 0.65 g (16 mmol) sodium hydroxide. Subsequently, 55 mL (0.8 mol) of 25% aqueous ammonia was added and the reaction mixture heated to reflux for 48 h. After cooling to rt the solid was filtered off and dissolved in 20 mL methylene chloride. 20 mL Methanol was added and the methylene chloride removed under reduced pressure. The precipitate was filtered off and dried in vacuo to yield 1.82 g (4.7 mmol, 58%) of 4′-(3-bromophenyl)-2,2′:6′,2″-terpyridine (**7b**) as a colorless solid. ^1^H NMR (500 MHz, CDCl_3_, δ in ppm): 7.40–7.43 (m, 3 H, Ar_tpy_-H^5,5″^/Ar_Ph_-H^5^), 7.61 (d, ^3^*J* = 8.8 Hz, 1 H, Ar_Ph_-H^6^), 7.87 (d, ^3^*J* = 7.5 Hz, 1 H, Ar_Ph_-H^4^), 7.93 (m, 2 H, Ar_tpy_-H^4,4″^), 8.08 (s, 1 H, Ar_Ph_-H^2^), 8.71 (d, ^3^*J* = 8.2 Hz, 2 H, Ar_tpy_-H^6,6″^), 8.74 (s, 2 H, Ar_tpy_-H^3′,5′^), 8.77 (d, ^3^*J* = 4.4 Hz, 2 H, Ar_tpy_-H^3,3″^). ^13^C NMR (125 MHz, CD_2_Cl_2_, δ in ppm): 118.6 (tpy^3′,5′^), 121.1 (tpy^3,3″^), 123.0 (C-Br), 124.0 (tpy^5,5″^), 126.0 (Ph^6^), 130.2 (Ph^4^), 130.6 (Ph^5^), 131.9 (Ph^2^), 136.9 (tpy^4,4″^), 140.7 (Ph^1^), 148.6 (tpy^4′^), 149.2 (tpy^6,6″^), 155.8 (tpy^2,2″^), 156.1 (tpy^2′,6′^). EI-MS (70 eV, *m/z*, %): 154 (12, [M−C_6_H_5_Br−C_5_H_4_N]^+^), 204 (8), 229 (15, [M−C_6_H_6_Br]^+^), 308 (100, [M−Br]^+^), 309/311 (25, [M−C_5_H_4_N]^+^), 387/389 (86, [M]^+^).

### Synthesis of 4′-[3-(5,5-dimethyl-1,3,2-dioxaborinan-2-yl)phenyl]-2,2′:6′,2″-terpyridine (**9b**)

584 mg (1.5 mmol) 4′-(3-Bromophenyl)-2,2′:6′2″-terpyridine (**7b**), 550 mg (5.6 mmol) dry potassium acetate and 444 mg (2.0 mmol) 5,5,5′,5′-tetramethyl-2,2′-di(1,3,2-dioxaborinan) (**8**) were dissolved in 12 mL dry DMSO. The reaction mixture was thoroughly degassed by the freeze-pump-thaw technique and subsequently 46 mg (63 μmol) [(dppf)PdCl_2_] was added and the mixture stirred for 6 h at 80 °C. The mixture was diluted with 100 mL toluene and the resulting organic layer washed with water (4 × 100 mL), dried over MgSO_4_ and filtered. The solvent was removed in vacuo and the residue dissolved in 20 mL methylene chloride. 20 mL Methanol was added and the methylene chloride removed under reduced pressure. The precipitate was filtered off and dried in vacuo to yield 497 mg (1.18 mmol, 78%) of 4′-[3-(5,5-dimethyl-1,3,2-dioxaborinan-2-yl)phenyl]-2,2′:6′,2″-terpyridine (**9b**) as a colorless solid. ^1^H NMR (500 MHz, CD_2_Cl_2_, δ in ppm): 1.09 (s, 6 H, CH_3_), 3.87 (s, 4 H, CH_2_), 7.41 (m, 2 H, Ar_tpy_-H^5,5″^), 7.57 (v tr, 1 H, Ar_Ph_-H^5^), 7.93 (m, 3 H, Ar_tpy_-H^4,4″^/Ar_Ph_-H), 8.00 (d, 1 H, Ar_Ph_-H), 8.33 (s, 1 H, Ar_Ph_-H^2^), 8.71 (m, 2 H, Ar_tpy_-H^6,6″^), 8.77 (m, 4 H, Ar_tpy_-H^3,3″^), 8.81 (s, 2 H, Ar_tpy_-H^3′,5′^). ^13^C NMR (125 MHz, CD_2_Cl_2_, δ in ppm): 21.6 (CH_3_), 31.8 (C(CH_3_)_2_), 72.3 (CH_2_), 118.7 (tpy^3′,5′^), 121.1 (tpy^3,3″^), 123.9 (tpy^5,5″^), 128.3 (Ph^6^), 129.4 (Ph^5^), 132.5 (Ph), 134.5 (Ph), 136.8 (tpy^4,4″^), 137.6 (Ph^1^), 149.1 (tpy^6,6″^), 150.3 (tpy^4′^), 155.9 (tpy^2,2″^), 156.1 (tpy^2′,6′^). EI-MS (70 eV, *m/z*, %): 257 (10, [tpy-C=CH]^••+^), 309 (34, [M−BO_2_C_5_H_9_]^+^), 335 (20, [M−C_5_H_10_O]^••+^), 406 (25, [M−CH_2_]^+^), 421 (100, [M]^+^).

### Synthesis of 3,3′-(perfluorocyclopent-1-ene-1,2-diyl)bis(2-methyl-6-(4-(2,2′:6′,2″-terpyridin-4′-yl)phenyl)benzo[*b*]thiophene) (**10a**)

400 mg (555 μmol) 3,3′-(Perfluorocyclopent-1-ene-1,2-diyl)bis(6-iodo-2-methylbenzo[*b*]thiophene) (**4**) was dissolved in 50 mL THF and 50 mL of 2 M aqueous sodium carbonate solution added. 515 mg (1.22 mmol) 4′-[4-(5,5-dimethyl-1,3,2-dioxaborinan-2-yl)phenyl]-2,2′:6′,2″-terpyridine (**9a**) was added and the reaction mixture thoroughly degassed by the freeze-pump-thaw technique. 140 mg (60 μmol) [Pd(PPh_3_)_4_] was added and the mixture stirred for 20 h at 80 °C. After cooling to rt the mixture was extracted with methylene chloride (3 × 50 mL), the combined organic layers washed several times with 1 M hydrochloric acid, dried over MgSO_4_ and filtered. After removal of the solvent under reduced pressure the crude yellow product was purified by column chromatography (basic alumina, eluting with THF) to yield 421 mg (389 μmol, 70%) of 3,3′-(perfluorocyclopent-1-ene-1,2-diyl)bis(2-methyl-6-(4-(2,2′:6′,2″-terpyridin-4′-yl)phenyl)benzo[*b*]thiophene) (**10a**) as a pale yellow solid. ^1^H NMR (500 MHz, CDCl_3_, δ in ppm): 2.33/2.59 (*ap/p*, s, 6 H, CH_3_), 7.42–7.47 (m, 5 H, Ar-H), 7.72–7.84 (m, 7 H, Ar-H), 7.94–8.12 (m, 10 H, Ar-H), 8.78–8.92 (m, 12 H, Ar-H). ^13^C NMR (125 MHz, THF-d_8_, δ in ppm): 14.5 (CH_3_), 118.1 (t), 118.8 (q), 120.3 (q), 120.5 (t), 120.7 (t), 123.7 (q), 123.8 (t), 124.1 (t), 127.4 (t), 127.5 (t), 127.6 (t), 136.5 (t), 137.0 (q), 137.6 (m, CF_2_), 139.3 (q), 141.08 (q), 141.15 (q), 143.9 (q), 149.1 (t), 149.2 (q), 156.0(q), 156.06 (q), 156.12 (q). ^19^F NMR (470 MHz, THF-d_8_, δ in ppm): −109.4 to −112.0 (m, 4 F, 3-/5-CF_2_), −132.7 to −134.2 (m, 2 F, 4-CF_2_). ESI-MS (positive mode, CHCl_3_/MeCN, *m/z*): 1083 [M]^+^. HRMS: calc. for [C_65_H_40_F_6_N_6_S_2_+H]^+^: 1083.27328; found: 1083.27181.

### Synthesis of 3,3′-(perfluorocyclopent-1-ene-1,2-diyl)bis(2-methyl-6-(3-(2,2′:6′,2″-terpyridin-4′-yl)phenyl)benzo[*b*]thiophene) (**10b**)

120 mg (167 μmol) 3,3′-(Perfluorocyclopent-1-ene-1,2-diyl)bis(6-iodo-methylbenzo[*b*]thiophene) (**4**) was dissolved in 20 mL THF and 10 mL of 2 M aqueous sodium carbonate solution added. 154 mg (367 μmol) 4′-[3-(5,5-dimethyl-1,3,2-dioxaborinan-2-yl)phenyl]-2,2′:6′,2″-terpyridine (**9b**) was added and the reaction mixture thoroughly degassed by the freeze-pump-thaw technique. 42 mg (18 μmol) [Pd(PPh_3_)_4_] was added and the mixture stirred for 20 h at 80 °C. After cooling to rt the mixture was extracted with methylene chloride (3 × 20 mL), the combined organic layers washed several times with 1 M hydrochloric acid, dried over MgSO_4_ and filtered. After removal of the solvent under reduced pressure, the crude yellow product was purified by column chromatography (basic alumina, eluting with THF) to yield 105 mg (97 μmol, 58%) of 3,3′-(perfluorocyclopent-1-ene-1,2-diyl)bis(2-methyl-6-(3-(2,2′:6′,2″-terpyridin-4′-yl)phenyl)benzo[*b*]thiophene) (**10b**) as a yellow solid which still contained impurities. ^1^H NMR (500 MHz, CDCl_3_, δ in ppm): 2.32/2.58 (*ap*/*p*, s, 6 H, CH_3_), 7.38–7.44 (m, 5 H, Ar-H), 7.73–7.83 (m, 7 H, Ar-H), 7.92–8.10 (m, 10 H, Ar-H), 8.71–8.88 (m, 12 H, Ar-H). MALDI-TOF (*m/z*): 1083 [M]^+^, 1106 [M+Na]^+^.

### Synthesis of (4′-(4-bromophenyl)-2,2′:6′,2″-terpyridine)ruthenium(III)chloride (**11**)

500 mg (1.29 mmol) 4′-(4-Bromophenyl)-2,2′:6′,2″-terpyridine (**7a**) was suspended in 10 mL methanol and 337 mg (1.29 mmol) ruthenium(III) chloride trihydrate added. The dark brown suspension was heated to reflux for 2 h and then cooled to rt. Filtration gave 423 mg (0.17 mmol, 55%) of the monoterpyridine complex **11** as a dark brown solid which was immediately used for the following reaction.

### Synthesis of [(**7a**)Ru(*tpy-diae-tpy*)Ru(**7a**)](PF_6_)_4_ (**12**)

150 mg (251 μmol) (4′-(4-Bromophenyl)-2,2′:6′,2″-terpyridine)ruthenium(III) chloride (**11**) was suspended in 10 mL ethanol and treated with 0.5 mL (3.9 mmol) *N*-ethylmorpholine. Subsequently, 136 mg (126 μmol) 3,3′-(perfluorocyclopent-1-ene-1,2-diyl)bis(2-methyl-6-(4-(2,2′:6′,2″-terpyridin-4′-yl)phenyl)benzo[*b*]thiophene) (**10a**, *tpy-diae-tpy*) was added and the mixture heated under reflux for 3 h. The binuclear complex [(**7a**)Ru(*tpy-diae-tpy*)Ru(**7a**)]^4+^ was precipitated by the addition of 200 mg (1.23 mmol) ammoniumhexafluorophosphate and separated by centrifugation. The resulting solid was purified by column chromatography (silica gel, eluting with acetonitrile/water/sat. aq KPF_6_ soln 95:4:1) to yield 133 mg (50 μmol, 40%) of [(**7a**)Ru(*tpy-diae-tpy*)ru(**7a**)](PF_6_)_4_ (**12**) as a dark red solid. ^1^H NMR (500 MHz, CD_3_CN, δ in ppm): 2.51/2.68 (*ap*/*p*, s, 6 H, CH_3_), 7.16–7.23 (m, 9 H), 7.43–7.49 (m, 9 H), 7.93–8.01 (m, 15 H), 8.14–8.17 (m, 7 H), 8.33–8.39 (m, 5 H), 8.64–8.72 (m, 9 H), 9.00–9.11 (m, 8 H). MALDI-TOF (*m/z*): 2207 [M−3 PF_6_]^+^, 2352 [M−2 PF_6_]^+^, 2497 [M−PF_6_]^+^. ESI-MS (positive mode, MeCN, *m/z*): 2497 ([M−PF_6_]^+^). ESI-MS (negative mode, MeCN, *m/z*): 2787 ([M+PF_6_]^−^) [19].

### General procedures for transition metal complexes of 3,3′-(perfluorocyclopent-1-ene-1,2-diyl)bis(2-methyl-6-(4-(2,2′:6′,2″-terpyridin-4′-yl)phenyl)benzo[*b*]thiophene) (**10a**)

3,3′-(Perfluorocyclopent-1-ene-1,2-diyl)bis(2-methyl-6-(4-(2,2′:6′,2″-terpyridin-4′-yl)phenyl)benzo[*b*]thiophene) (**10a**) was suspended in methanol. FeCl_2_ × 4 H_2_O, CoCl_2_, [Ni(acac)_2_] or Zn(F_3_CSO_3_)_2_ was added and the resulting suspension stirred vigorously until all solid had dissolved. The formation of the iron(II) and cobalt(II) complexes can be traced by the color change from colorless to blue (iron(II)) and red (cobalt(II)), respectively.

## Supporting Information

Supporting information contains UV–vis-spectra of [Co^2+^@**10a**], [Ni^2+^@**10a**], ^1^H NMR-spectra of **7b**, **9b**, **10a**, **10b** and **12**, ^13^C NMR-spectra of **7b**, **9b** and **10a**, the ^19^F NMR-spectrum of **10a** and the simulated ESI-MS-spectra of **12**.

File 1 NMR-, UV- and MS-spectra.
