# Supplementary material for: A perfluorocyclopentene based diarylethene bearing two terpyridine moieties – synthesis, photochemical properties and influence of transition metal ions
Source: Beilstein J Org Chem. 2010 May 26;6:53. doi: 10.3762/bjoc.6.53 (PMC2900936; doi:10.3762/bjoc.6.53)
Supplement: File 1 — NMR-, UV- and MS-spectra. [file Beilstein_J_Org_Chem-06-53-s001.pdf]

Supporting Information
for

A perfluorocyclopentene based diarylethene bearing two terpyridine moieties – synthesis, photochemical properties and influence of transition metal ions

Falk Wehmeier[2] and Jochen Mattay[*1]

Address: [1]Department of Chemistry, Organic Chemistry 1, Bielefeld University, P. O. box 10 01 31, D-33501 Bielefeld, Germany and [2]Institut für Chemie, Humboldt-Universität zu Berlin, Brook-Taylor-Str. 2, D-12489 Berlin, Germany

Email: Jochen Mattay - oc1jm@uni-bielefeld.de

* Corresponding author

NMR-, UV- and MS-spectra

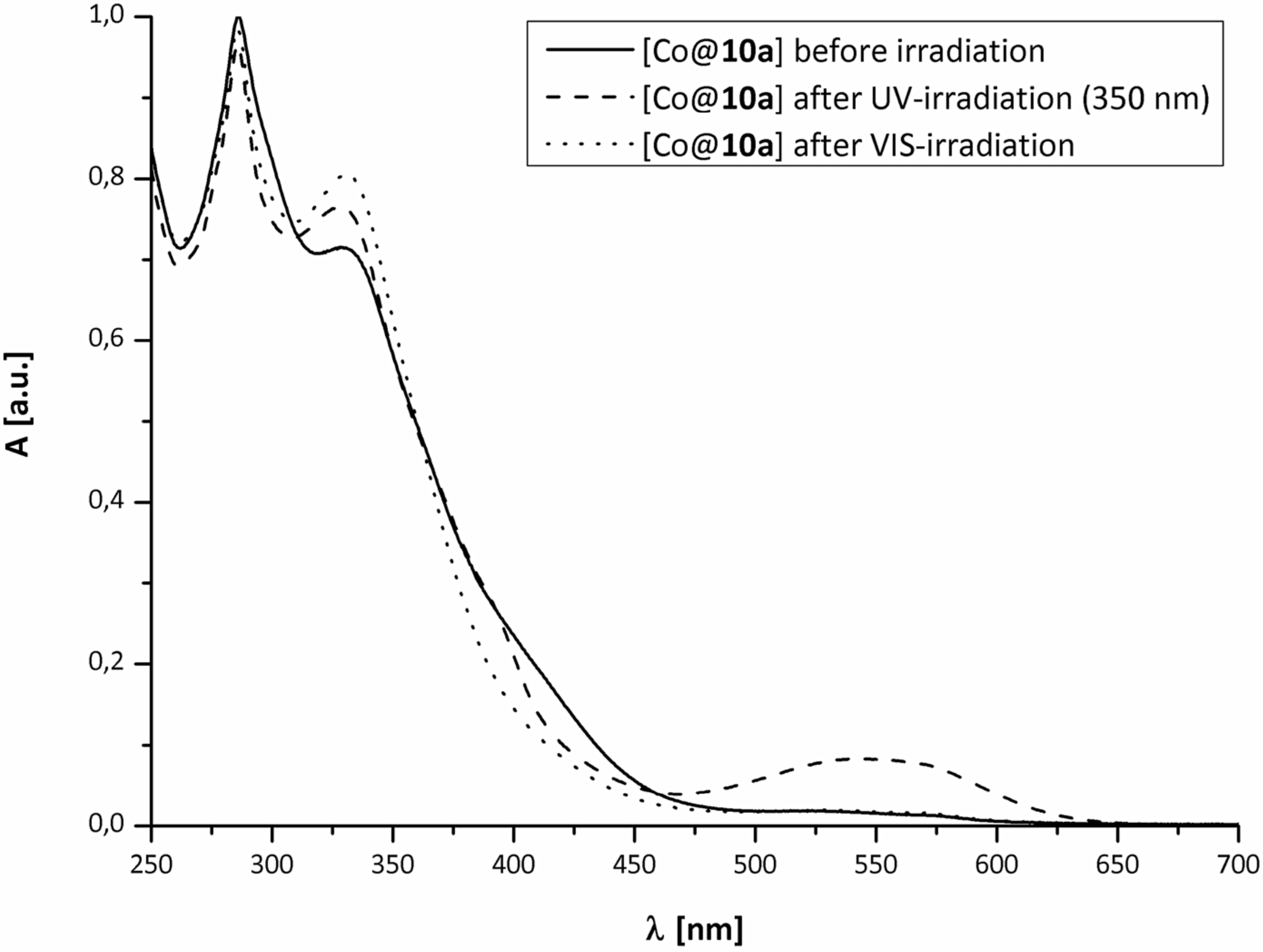

[Co@10a] before irradiation
[Co@10a] after UV-irradiation (350 nm)
[Co@10a] after VIS-irradiation
A [a.u.]
λ [nm]
1,0
0,8
0,6
0,4
0,2
0,0
250
300
350
400
450
500
550
600
650
700

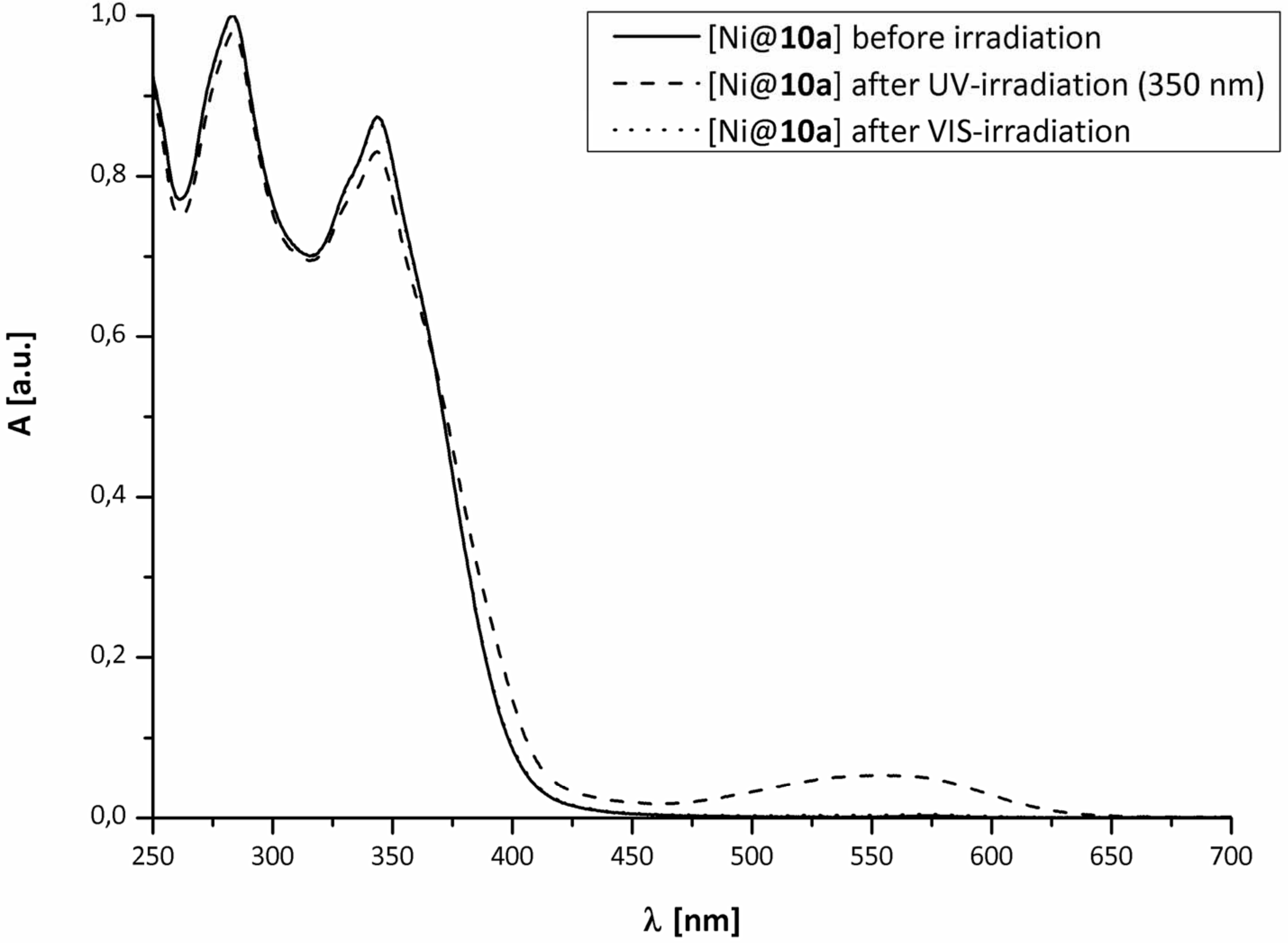
[Ni@**10a**] before irradiation
[Ni@**10a**] after UV-irradiation (350 nm)
[Ni@**10a**] after VIS-irradiation
A [a.u.]
1,0
0,8
0,6
0,4
0,2
0,0
250
300
350
400
450
500
550
600
650
700
λ [nm]

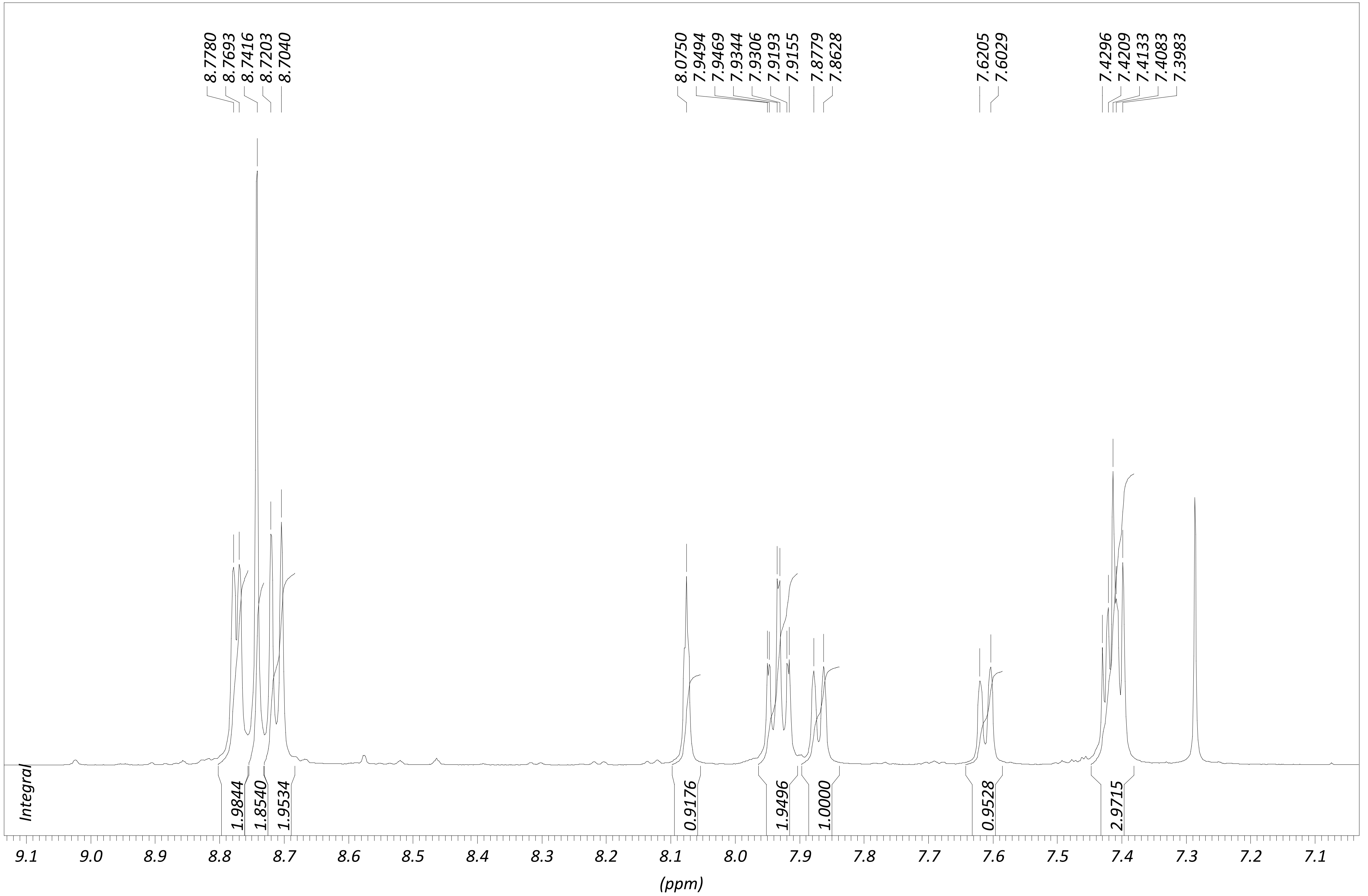

1H-NMR-spectrum of 7b

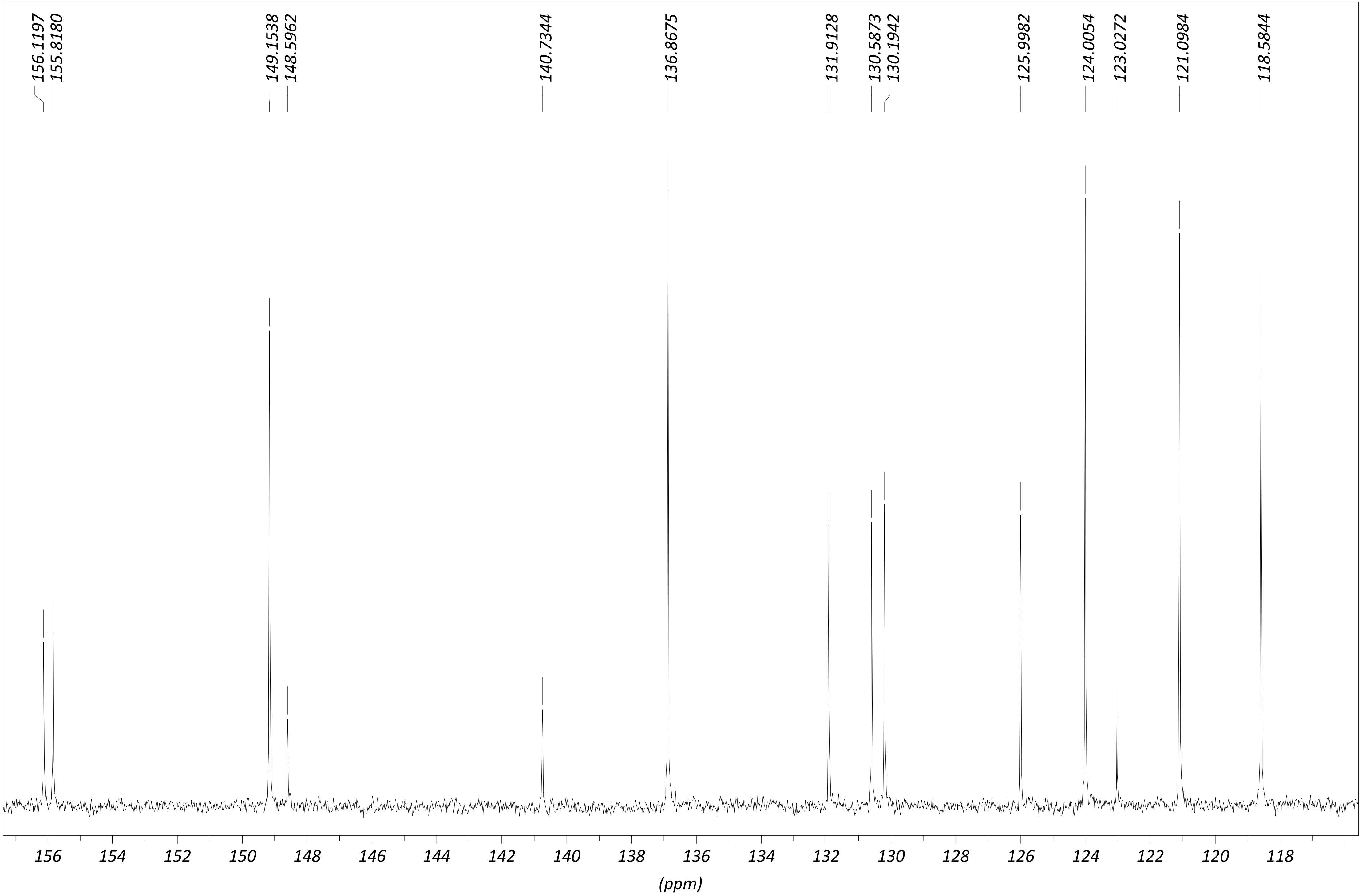

13C-NMR-spectrum of 7b

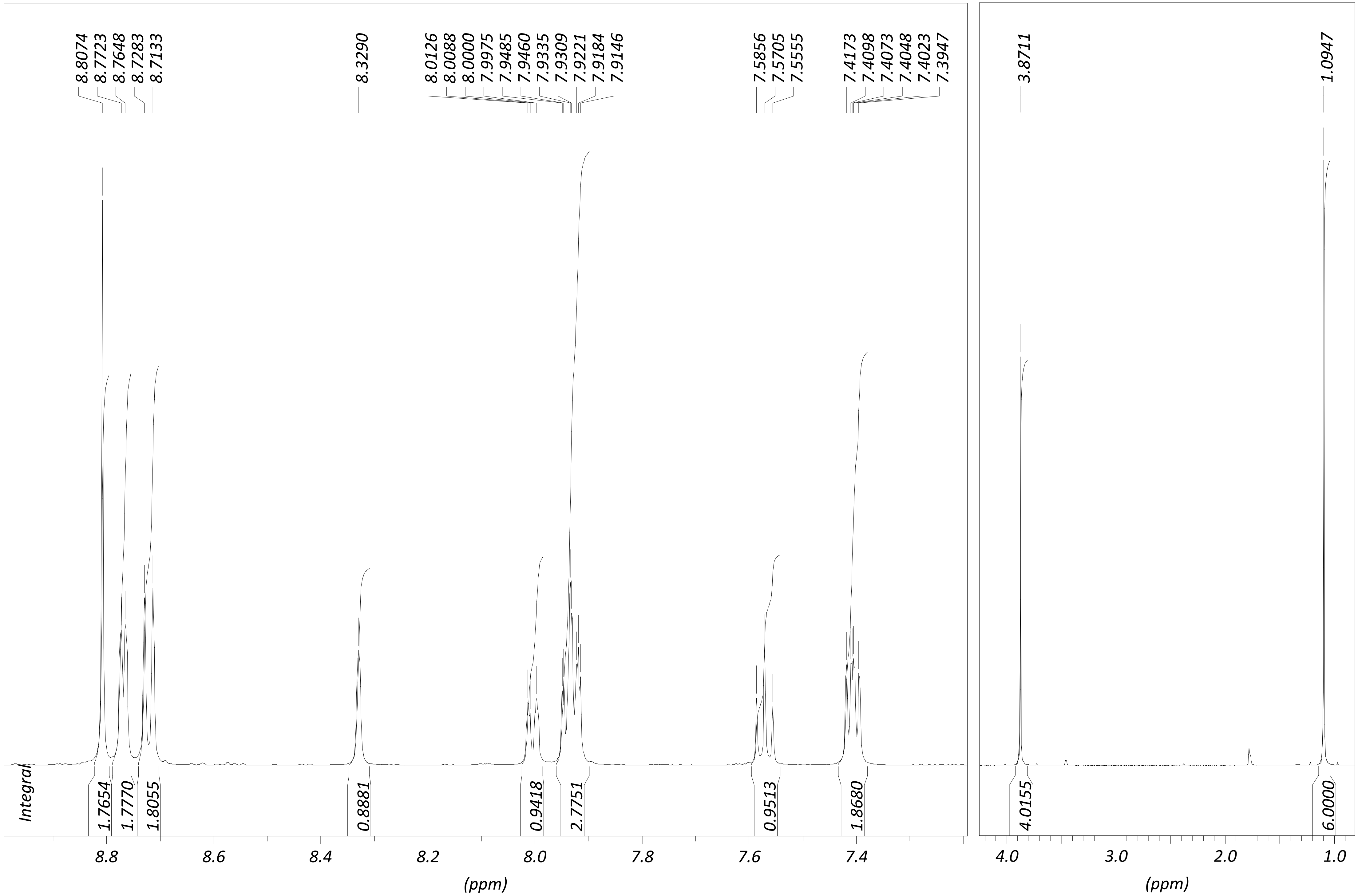

1H-NMR-spectrum of 9b

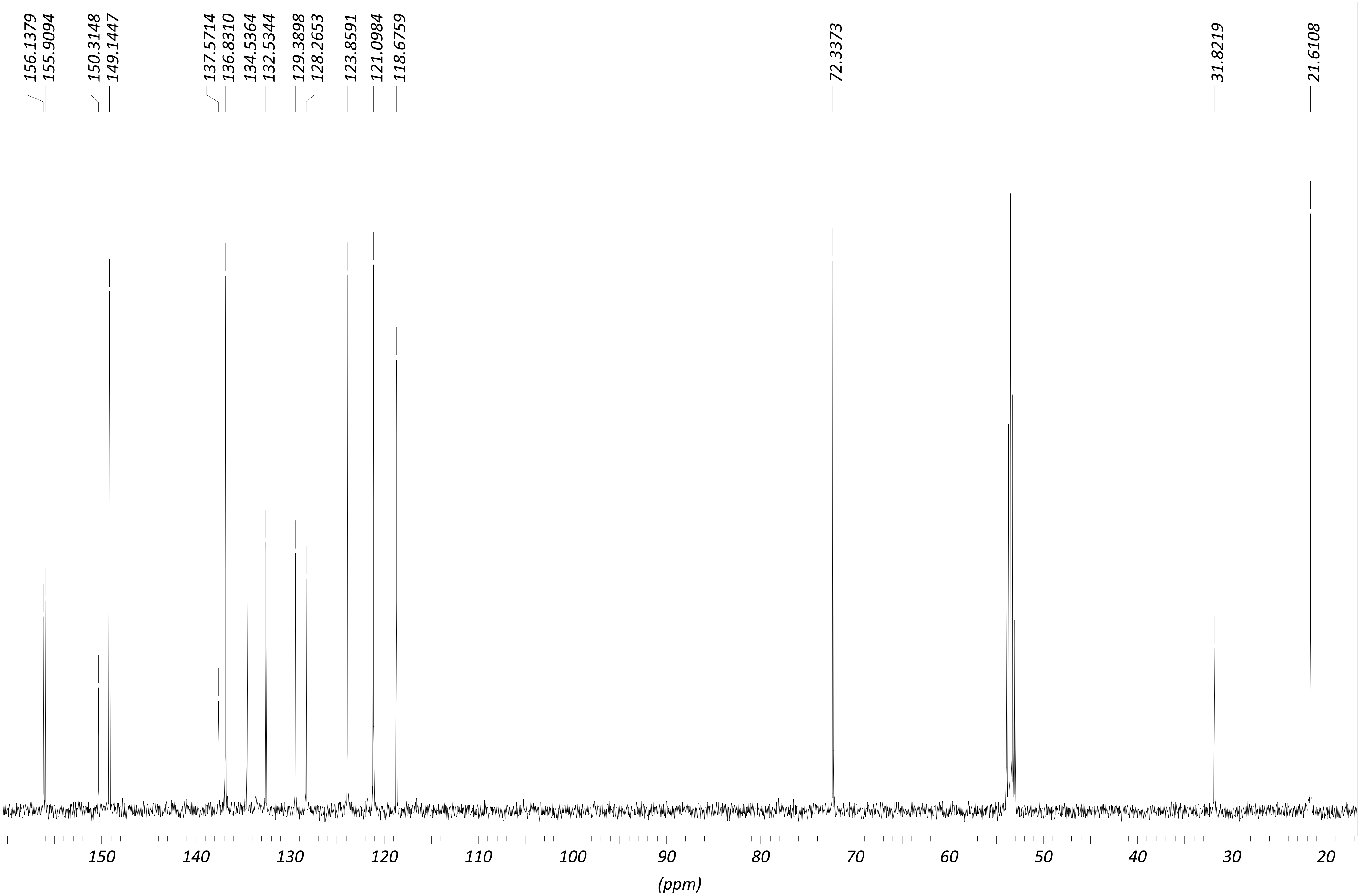

13C-NMR-spectrum of 9b

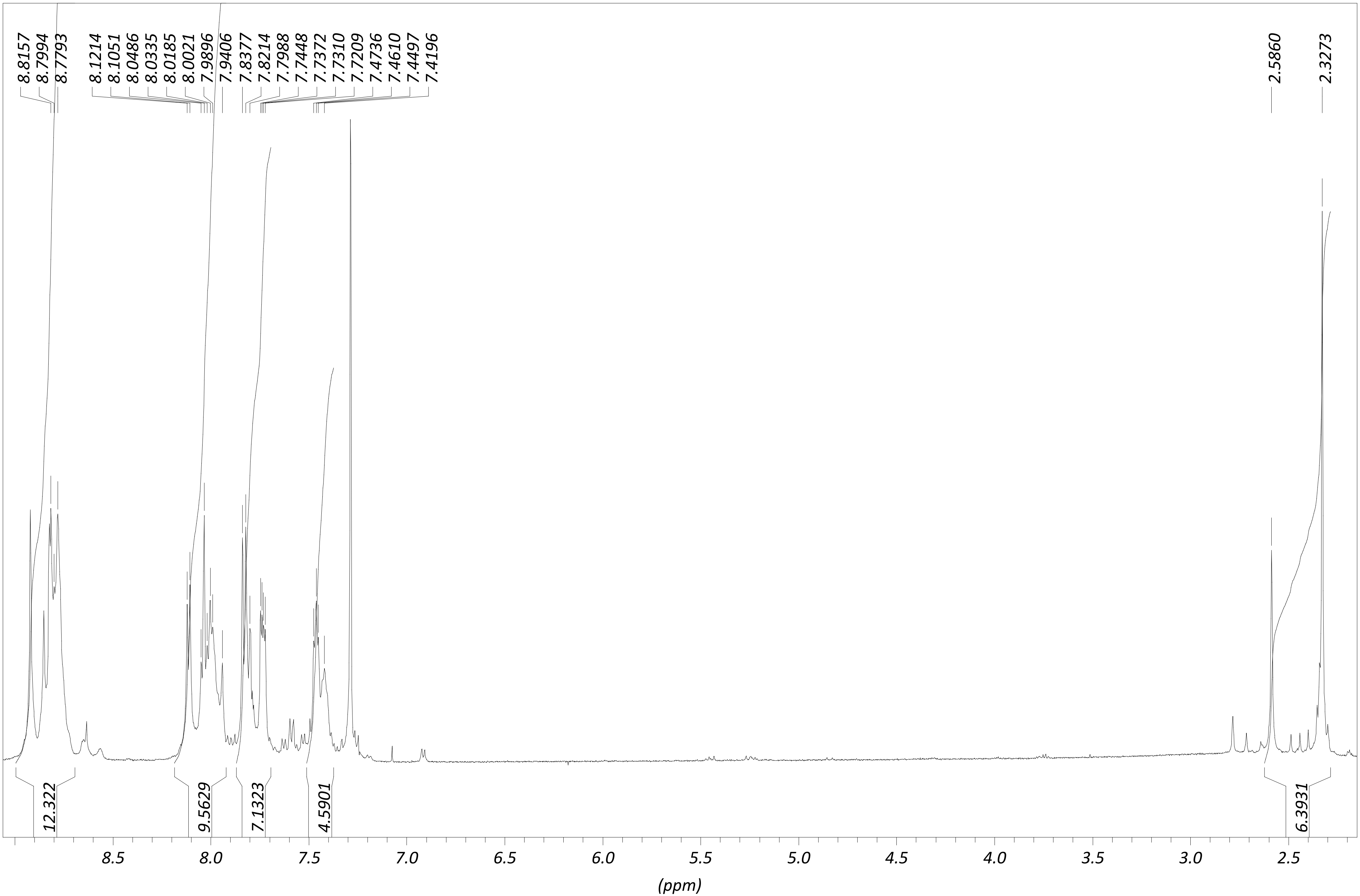

1H-NMR-spectrum of 10a

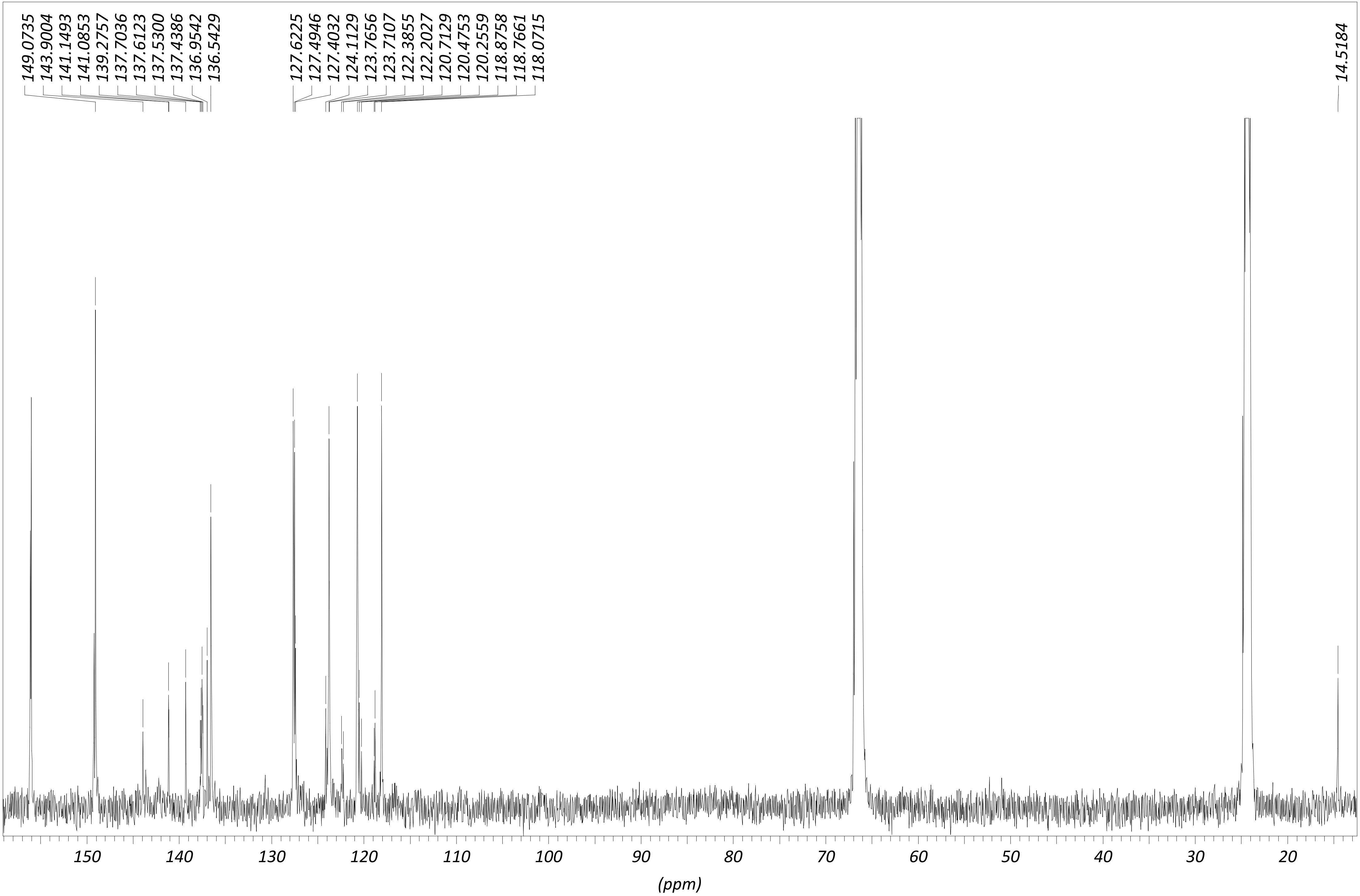

13C-NMR-spectrum of 10a

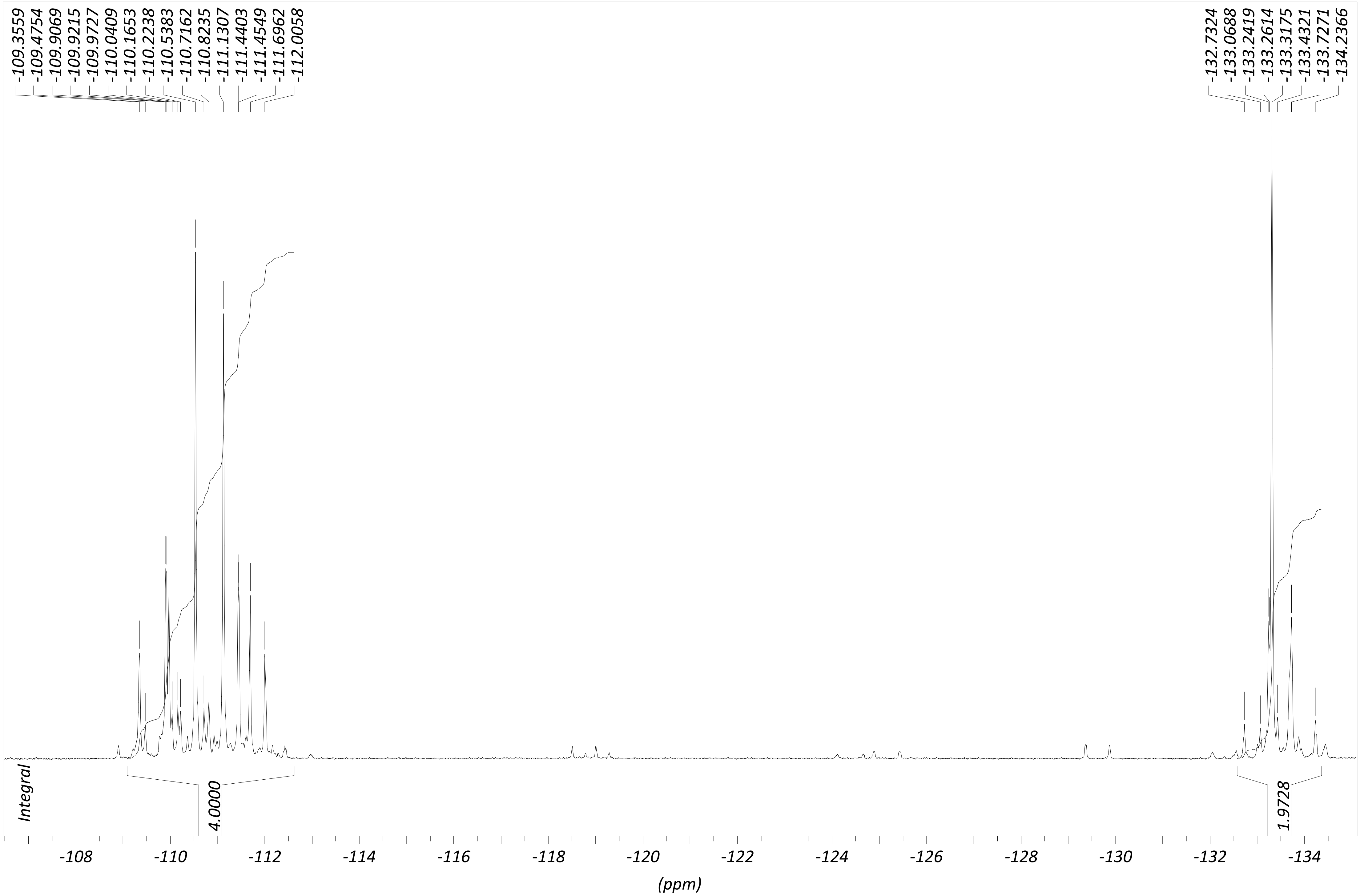

19F-NMR-spectrum of 10a

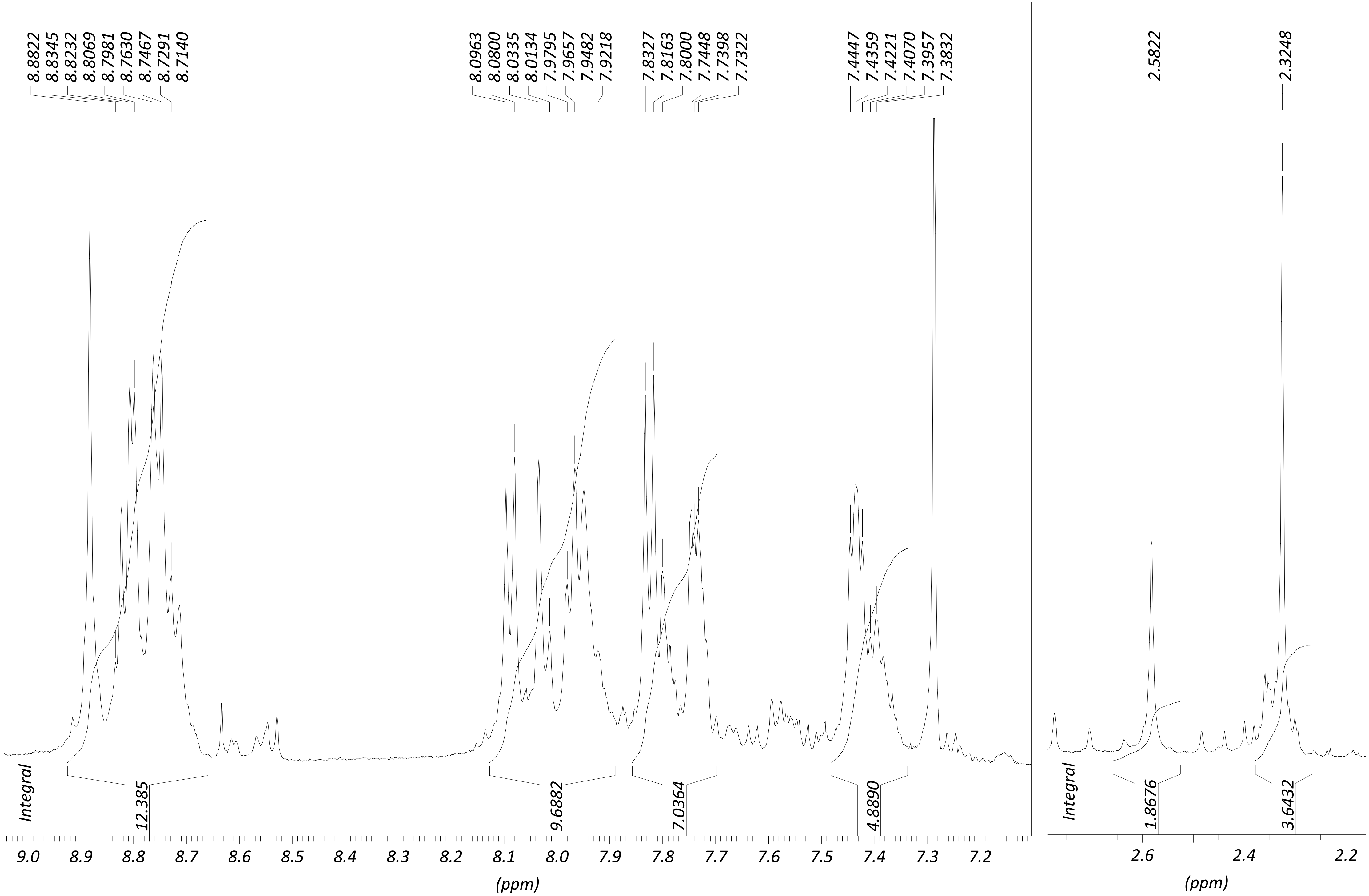

1H-NMR-spectrum of 10b

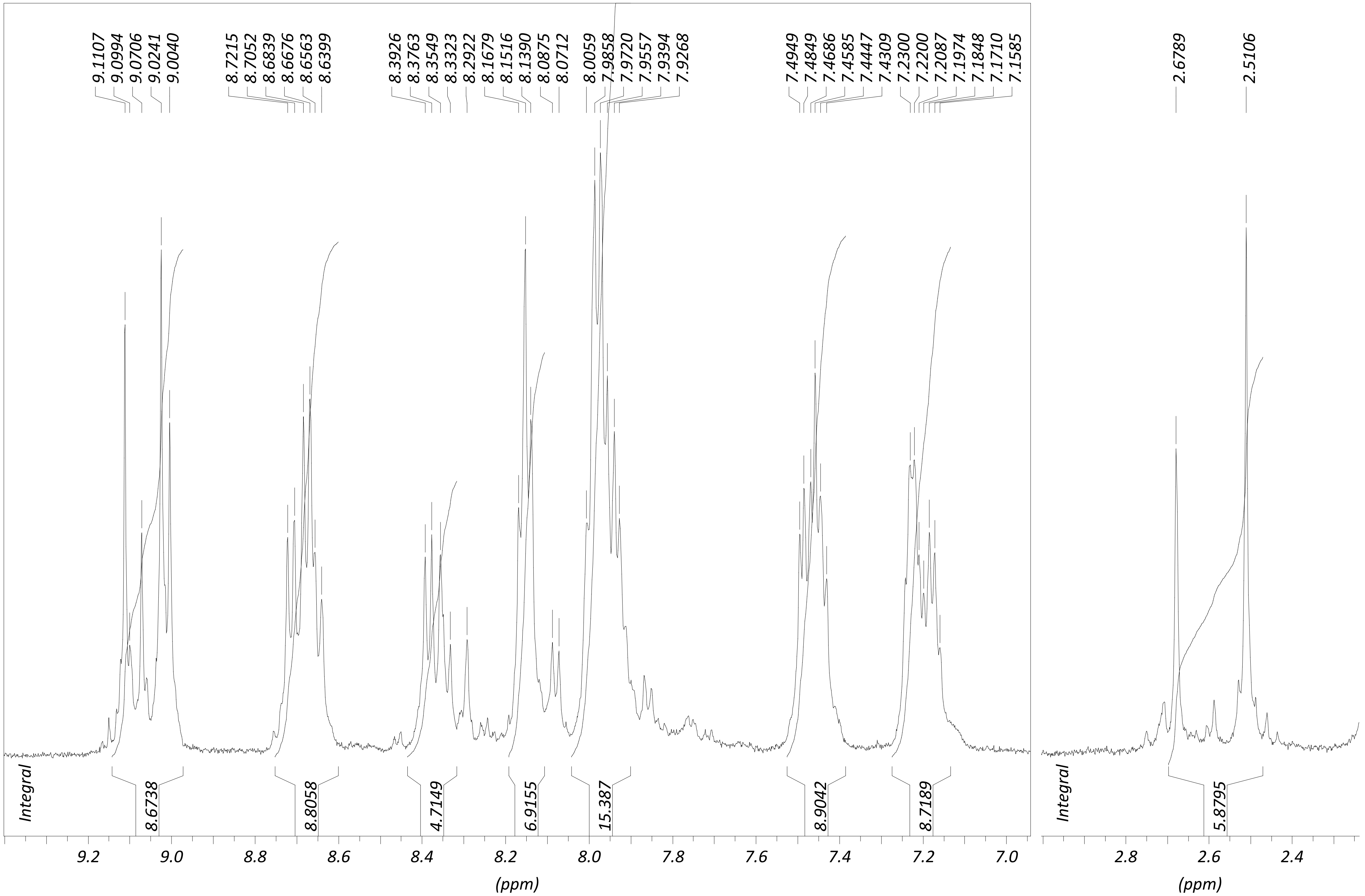

1H-NMR-spectrum of 12

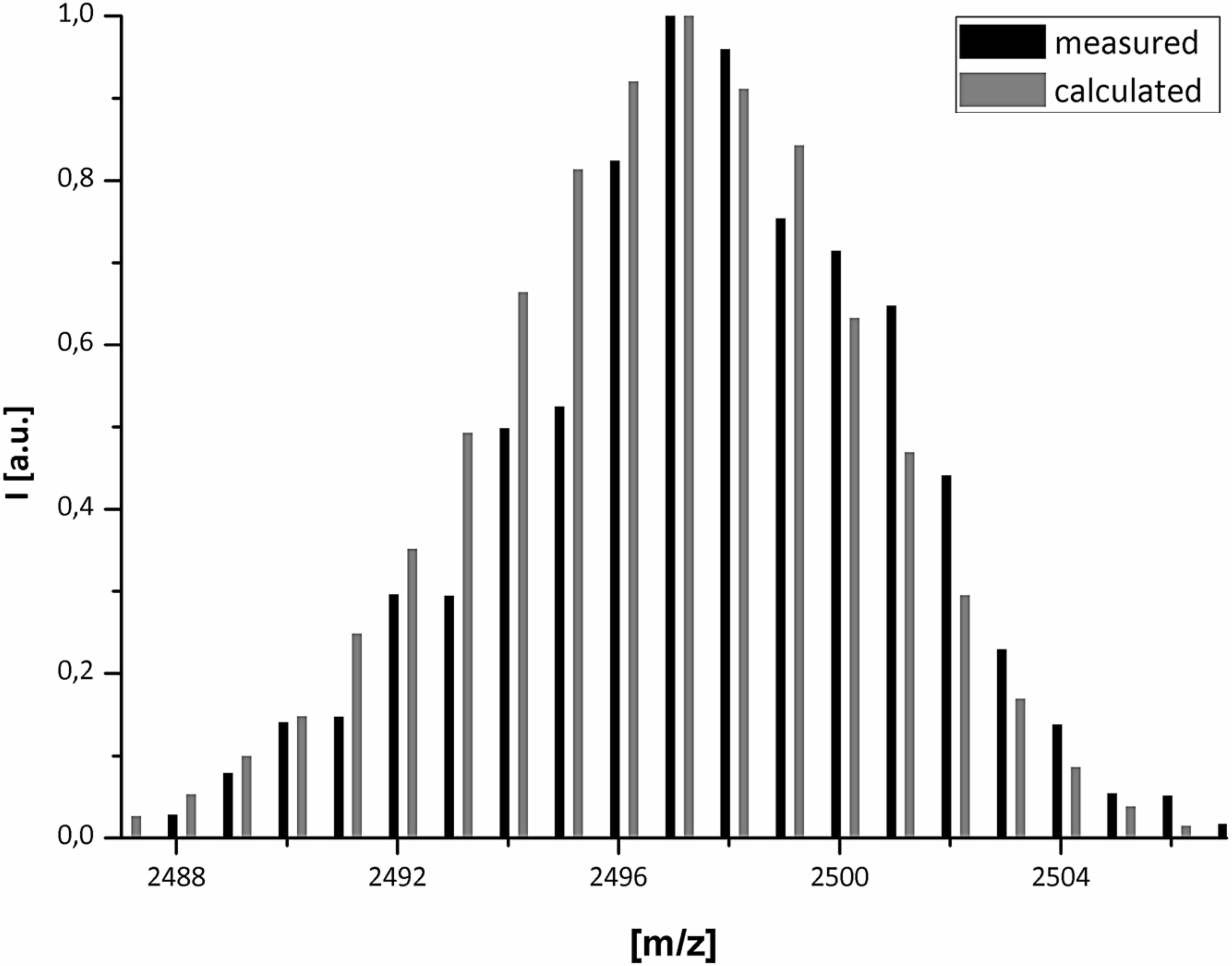
1,0
0,8
0,6
0,4
0,2
0,0
I [a.u.]
2488
2492
2496
2500
2504
[m/z]
measured
calculated

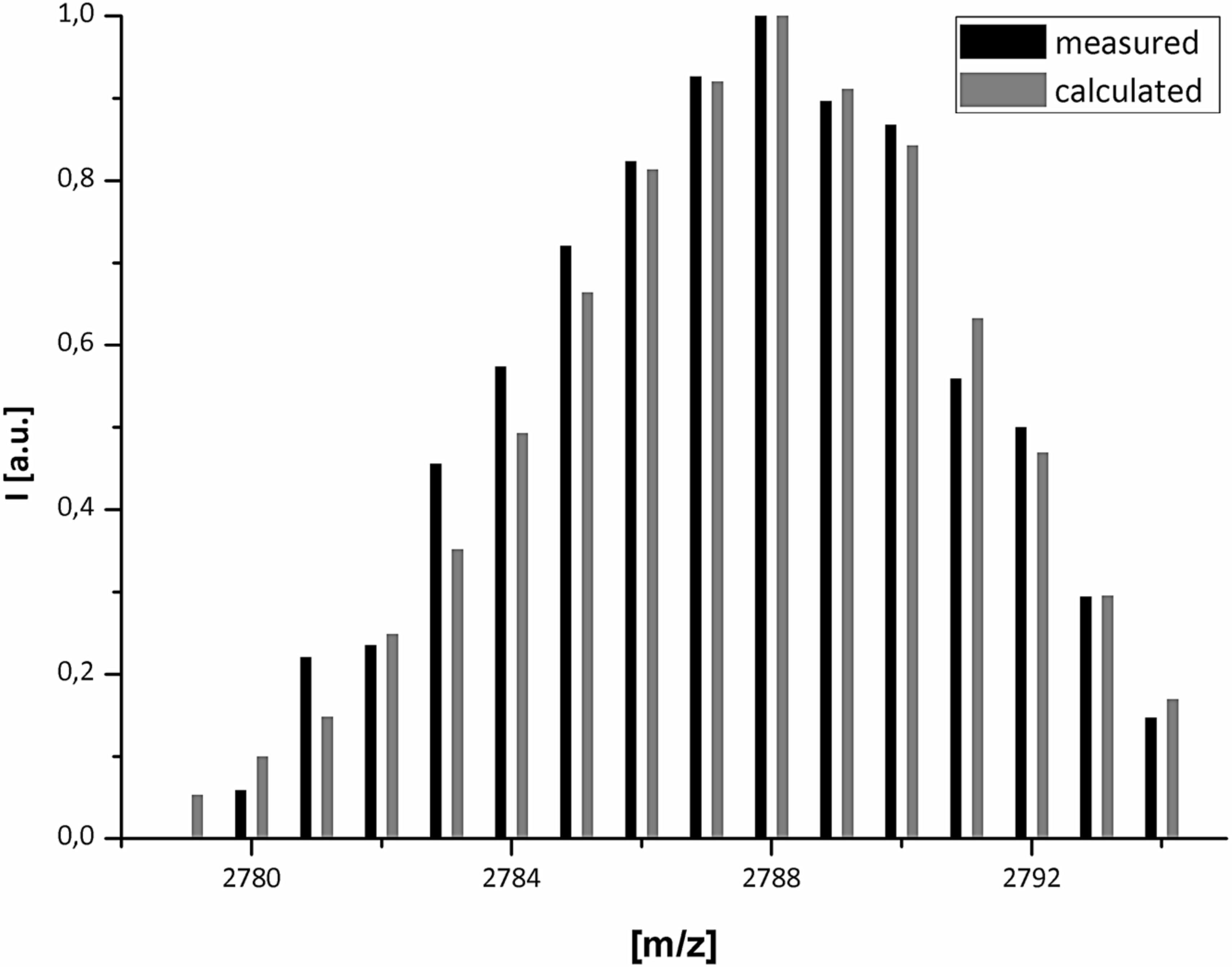
1,0
0,8
0,6
0,4
0,2
0,0
I [a.u.]
2780
2784
2788
2792
[m/z]
measured
calculated
